# Network processes involved in the mediation of short-term habituation in *Aplysia*: contribution of intrinsic regulation of excitability and synaptic augmentation

**DOI:** 10.3389/fnint.2014.00015

**Published:** 2014-02-13

**Authors:** Thomas M. Fischer, Daniel A. Jacobson, Kristin Demorest-Hayes

**Affiliations:** Department of Psychology, Wayne State UniversityDetroit, MI, USA

**Keywords:** interneuron, excitability, inhibition, reflex, mechanoreceptor, network

## Abstract

Short-term habituation (STH) is the decrease in behavioral responding observed during repeated stimulation at regular intervals. For siphon-elicited siphon withdrawal in *Aplysia* (S-SWR), we previously showed that the amplitude of responses measured in LFS-type siphon motor neurons (LFS MNs) during training is dependent on the stimulus interval used and is training-site specific. The major source of excitation from siphon stimulation onto the LFS MNs comes from the L29 interneurons. Here we examined the role of the L29s in STH by addressing two questions:

(1) What are the relative contributions of intrinsic regulation of excitability and network inhibition on L29 activity during STH training?

By activating L29s with intracellular current injection, we found that intrinsic changes in excitability occur, but only at short training intervals (1 s). We also demonstrated that network inhibition is not required for regulating L29 responses during training, indicating that any expression of inhibition is redundant to the excitability changes.

(2) How does L29 synaptic plasticity contribute to the maintenance of training site-specificity exhibited in LFS MNs?

When training stimuli are delivered 1 s apart [1 s, interstimulus interval (ISI)], L29 responses decrease in both stimulated (trained) and un-stimulated (untrained) pathways, yet site-specificity of training is maintained in the LFS MNs. Our results suggest that activity-dependent synaptic facilitation (augmentation; AUG) expressed by the L29s acts to compensate for the decreased activity in the untrained pathway. First, we demonstrated that the L29-LFS synapse exhibits significant AUG with L29 activation at a 1 s ISI. Second, we showed that the induction of AUG prevents the reduction in siphon-evoked LFS responses that is otherwise observed with decreased L29 activity. Collectively, our results support a role for the L29s in regulating network dynamics during STH training, but only at rapid (1 s ISI) training intervals.

## INTRODUCTION

Dynamic processes within neural networks underlie the capacity for organisms to utilize information to adaptively regulate behavior. These processes include intrinsic changes resulting from activity, and/or extrinsic regulation such as synaptic inhibition or neuromodulation. Further, these processes often occur concurrently at multiple sites in a network (Frost et al., 1988; Chandler and Grossberg, 2012; Garrido et al., 2013). This inherent complexity of neural networks dictates that any understanding of neural mechanisms underlying behavioral plasticity must not only catalog the underlying cellular changes, but also describe how these processes interact to yield a net behavioral output. We have examined short-term habituation (STH, also referred to as “within-session” habituation: Thompson and Spencer, 1966; Thompson, 2009) as a means to understand how a simple neural network dynamically adjusts behavioral responsiveness to match sensory information from the environment. STH refers to the decrement in responding observed during training with regularly spaced stimuli, with the rate of decrement determined by the interval between stimuli (ISI). We utilize the siphon withdrawal response (SWR) in the marine mollusk *Aplysia californica* as an experimental model system, which has proven useful in relating cellular processes directly to diverse forms of behavioral regulation (Carew and Kandel, 1973; Wright and Carew, 1995; Cohen et al., 1997; Fischer et al., 1997; Sutton et al., 2001; Philips et al., 2011). In this preparation, the effects of habituation training are restricted to the site of training, even when a second “untrained” site resides a few centimeters apart on the siphon. This site-specificity of training is taken as evidence that sensory afferents represent the primary locus of change with habituation (Frost et al., 1997; Ezzeddine and Glanzman, 2003).

**FIGURE 1:**
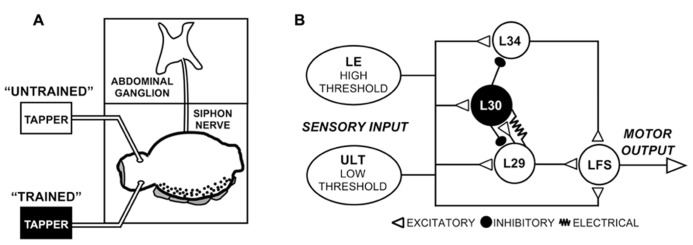
**Experimental preparation and network schematic.** **(A)** Semi-intact preparation used in these experiments, consisting of the siphon/mantle, the abdominal ganglion, and the siphon nerve that connects them. Tapping the siphon with a mechanical stimulator evokes sensory input to the withdrawal network. In some experiments (**Figure 2**), two tappers were used as illustrated to examine site-specificity of training, as illustrated. **(B)** Simplified version of the SWR network emphasizing the major elements in the reflex circuit. While only one cell of each type is shown, cells are multiply represented in the network. Two parallel sensory pathways from the siphon provide excitatory input, the lower threshold ULTs and the higher threshold LEs. Figure modified from Fischer et al. (2011).

Siphon MNs receive input both directly from sensory neurons as well as from a restricted set of identified interneurons such as the L29-type excitatory interneurons (**Figure 1B**; Hawkins et al., 1981; Cleary et al., 1995; Frost and Kandel, 1995). Depending on the stimulus, interneurons are estimated to provide around 75% of the net evoked input to siphon MNs (Trudeau and Castellucci, 1992), with single L29 neurons (out of five total) accounting for an average of 15% of this input (Fischer and Carew, 1993). Two parallel sensory pathways can be distinguished based on their stimulus thresholds (Byrne et al., 1978b; Frost et al., 1997; Illich and Walters, 1997; Calin-Jageman and Fischer, 2007). The well-characterized LE mechanoreceptors have cell bodies within the abdominal ganglion (Castellucci et al., 1970), and have stimulus activation thresholds higher than that required to evoke responses in MNs (Frost et al., 1997; Illich and Walters, 1997; Calin-Jageman and Fischer, 2007). The somata of the lower-threshold sensory neurons have yet to be identified, hence we refer to these as the unidentified low threshold (ULT) mechanoreceptors (Calin-Jageman and Fischer, 2007; Fischer et al., 2011).

Our studies on STH have used low-intensity siphon taps that only activate the ULTs. We found that changes in the ULTs alone can account for SWR network responses during habituation training at a 30 s ISI, an interval commonly used in studies of habituation in this preparation (Castellucci et al., 1970; Pinsker et al., 1970; Carew et al., 1972; Peretz et al., 1976; Castellucci et al., 1978; Rankin and Carew, 1987; Falk et al., 1993; Ezzeddine and Glanzman, 2003; Fischer et al., 2011). First, STH-induced regulation of both the L29 and LFS MN activity was training site-specific (Fischer et al., 2011), implicating sensory input as the key site of change (Frost et al., 1997; Ezzeddine and Glanzman, 2003). Second, there is a direct linear relationship between siphon-evoked ULT activity and responses in both L29 interneurons and LFS-type siphon MNs with single (non-habituating) stimuli. During habituation training, the ratio of ULT to LFS activity remained constant as overall network activity adjusted to a reduced asymptotic level. Together, these observations indicate that changes in the activity level of the ULTs alone can account for the observed changes in the network.

Two observations indicated that additional mechanisms beyond the regulation of ULT activity were involved in network regulation at a more rapid training interval (1 s ISI). First, the rate of decrement during training was faster for both the L29s and LFS MNs than that of ULT activity. Consistent with this, the ratio of ULT activity to LFS responses during training was non-linear, indicating the involvement of processes in addition to decreased sensory activity. Second, site-specificity of training was not preserved in the L29s, which exhibited a significant decrease in activity in response to stimulation of an untrained pathway. Despite this, site-specificity of training was preserved in the LFS MNs (Fischer et al., 2011). This raises the question as to how evoked MN activity remains constant when the activity of a major source of excitation is reduced.

Here, we focused primarily on the role of the L29 interneurons in regulating SWR network activity at rapid (1 s ISI) training intervals. L29s are of particular interest both because they are a major source of excitatory input to LFS MNs (Fischer and Carew, 1993; Frost and Kandel, 1995) and they are a principal target for the L30 inhibitory interneurons that regulate the SWR following tactile stimulation (**Figure 1B**, Hawkins et al., 1981; Fischer and Carew, 1995; Frost and Kandel, 1995; Calin-Jageman and Fischer, 2003a). First, we examined whether L29 excitability (or intrinsic plasticity: Zhang and Linden, 2003) is regulated with STH. We describe excitability changes at a 1 s ISI that are absent at a 30 s ISI, and suggest that this form of plasticity could account for both the accelerated rate of change observed in the network at a 1 s ISI as well as the lack of site-specificity of training observed in the L29s. Second, we explored the hypothesis that augmentation (AUG), a form of short-term synaptic enhancement expressed by the L29s, can act to maintain site-specificity of training. We show that training at a 1 s ISI induces AUG in the L29s, and that the induction of AUG can compensate for reduced L29 activity. Collectively, our results help confirm that that regulation of ULT activity is a primary underlying mechanism of STH at a 30 s ISI, with additional processes intrinsic to the L29s contributing at a 1 s ISI.

## MATERIALS AND METHODS

### ANIMALS

Wild-caught adult *A. Californica* (150–400 g) were acquired from Marinus Incorporated (Long Beach, CA, USA). Animals were housed in a custom 600-L aquarium with circulating Instant Ocean artificial seawater (ASW; Aquarium Systems, Mentor, OH, USA), maintained on a 12-h light:dark cycle, and fed dried seaweed (nori) five times per week.

### EXPERIMENTAL PREPARATION

Experiments were conducted with semi-intact preparations consisting of the siphon, mantle shelf, siphon nerve, and abdominal ganglion (**Figure 1A**). Animals were anesthetized by injection of isotonic MgCl_2_ into the body cavity at a volume of 0.5 ml/g of body weight. The siphon and mantle, along with the siphon nerve and connected abdominal ganglion, were then dissected from the animal. The preparation was transferred to a two-chambered recording dish coated with Sylgard (Dow Corning, Midland, MI, USA), with the abdominal ganglion placed in a separate compartment from the rest of the preparation. The abdominal ganglion was pinned ventral side up using minute pins, and the mantle was secured dorsal side up using 26_G_ hypodermic needles. The siphon was not restrained. Throughout the experiment, the siphon was continuously perfused with ASW through a cannula placed in the siphon artery. At least 45 min of post-dissection recovery time was allowed prior to physiological recordings.

### INTRACELLULAR RECORDINGS

To facilitate intracellular recordings, the sheath covering the left hemi-ganglion was surgically removed to expose the underlying neurons. Neurons were impaled with glass microelectrodes filled with 3 M KCL (resistance 6–12 mΩ). Potentials were amplified on a Dagan IX2-700 amplifier (Dagan, Minneapolis, MN, USA) and then digitized (Powerlab 8SP) for computer analysis using the Chart software package (ADInstruments, Colorado Springs, CO, USA).****Individual cell types were identified using established criteria (Hawkins et al., 1981; Frost and Kandel, 1995).****LFS MNs were identified based on their position within the abdominal ganglion, and by observing the characteristic siphon movements produced during their intracellular activation (Hickie and Walters, 1995). No distinction was made between LFS MN sub-types. L29 interneurons were identified based on their size and position, their recruitment of recurrent inhibition during intracellular activation, and their characteristic response to siphon tap (Hawkins et al., 1981; Fischer and Carew, 1993; Frost and Kandel, 1995). L29 interneurons were excluded from analysis if they responded with fewer than five action potentials to a siphon tap. This likely biases our sample towards the L29-A sub-type, which exhibit a greater siphon tap response than the L29-B sub-type (Fischer and Carew, 1995; Fang and Clark, 1996).

### EXPERIMENTAL PROCEDURES

In these experiments, training was accomplished either through tactile stimulation via siphon taps or through activating individual neurons directly with intracellular current injection. The siphon was tapped with glass probes attached to a stimulator-driven mechanical relay (**Figure 1A**; Fischer and Carew, 1993). The intensity of the tap (approximately 4 g/mm^2^) was similar to that we have used in previous experiments, which we have shown falls below the intensity threshold required to activate the LE sensory neurons when the siphon is not pinned to a substrate (Illich and Walters, 1997; Calin-Jageman and Fischer, 2007; Fischer et al., 2011). L29 neurons were allowed to remain at rest. Siphon tap-evoked activity was measured by counting the number of spikes within a 500 ms period following the first evoked spike, as we have done previously (Fischer and Carew, 1995; Fischer et al., 2000; Fischer et al., 2011). Current was injected in the L29s through the same electrode used for recording, and consisted of 300 ms depolarizing square wave pulses, the approximate duration of the underlying depolarizing potential observed following siphon tap. The amplitude of the current was adjusted to produce 5–7 spikes, a number typically observed following siphon tap under our experimental conditions (Fischer et al., 2011). LFS MNs were hyperpolarized to -85 mV, approximately 40 mV below rest. At this holding potential, inhibitory synaptic potentials would be reversed, so measures of the tap-evoked complex PSP would consist of a combination of IPSPs and EPSPs. The complex PSP measure was obtained by calculating the area underneath the initial 500 ms of the siphon-evoked response (in μV ms) using Chart software package. This measurement accounts for changes in both response amplitude and duration of the complex PSP (Fischer and Carew, 1993). The amplitude of monosynaptic EPSPs of the L29 to LFS synapse was also measured using Chart.

To measure siphon tap-evoked ULT sensory neuron activity, we obtained extracellular recordings *en passant *from the siphon nerve with a monopolar suction electrode (A-M Systems, Carlsborg, WA, USA) using techniques described in detail elsewhere (Calin-Jageman and Fischer, 2007; Fischer et al., 2011). Briefly, the abdominal ganglion was placed under anesthesia with isotonic MgCl_2_ to isolate afferent activity (Hickie et al., 1997; Calin-Jageman and Fischer, 2007). A small portion of the nerve was aspirated into the electrode using negative pressure. Signals were amplified using a BioAmp CF, high-pass filtered at 10 Hz, low-pass filtered at 100 Hz, and then digitized (Powerlab 8SP) for computer analysis using the Chart software package (all from ADInstruments, Colorado Springs, CO, USA). As a quantitative measure, we first used Chart to compute the absolute value of the tap-evoked response measured from the nerve, which incorporates both positive and negative deflections of the complex evoked waveform (an example of this transform is shown in Fischer et al., 2011). We then determined the integral of the absolute value (in μV ms) for 300 ms following the onset of the evoked response, which is the approximate duration of the evoked response following a siphon tap.

In some experiments, we blocked inhibition within the SWR circuit through bath administration of 100 μm curare dissolved in ASW (D-tubocuranine; Sigma-Aldrich, St. Louis, MO, USA). Acetylcholine binding to nicotinic-like receptors is a major form of inhibitory neurotransmission in the central ganglia of *Aplysia *(Tauc and Gerschenfeld, 1962; Kehoe, 1972; Segal and Koester, 1982; Trudeau and Castellucci, 1993; Storozhuk and Castellucci, 1999), including from the L30 inhibitory interneurons (Calin-Jageman and Fischer, 2003a). Incubation with curare began at least 10 min before data collection and continued throughout the experiment. The effectiveness of administration was confirmed by observing the general increase in excitability of neurons with the blockage of inhibition (Trudeau and Castellucci, 1993; Lieb and Frost, 1997; Calin-Jageman and Fischer, 2003a).

Our standard experiment consisted of a (1) baseline measure; (2) training through repeated taps or current injections; and (3) a recovery measure. Training always began 5 min following the baseline measure, and the recovery measure was always obtained 5 min post-training. A 5 min interval was used based on previous observations that this interval does not result in a decrease of tap-evoked afferent activity across five consecutive stimuli (Calin-Jageman and Fischer, 2007). Training consisted of 30 taps or current injections delivered at either a 1 or 30 s ISI.

### DATA ANALYSIS

**FIGURE 2:**
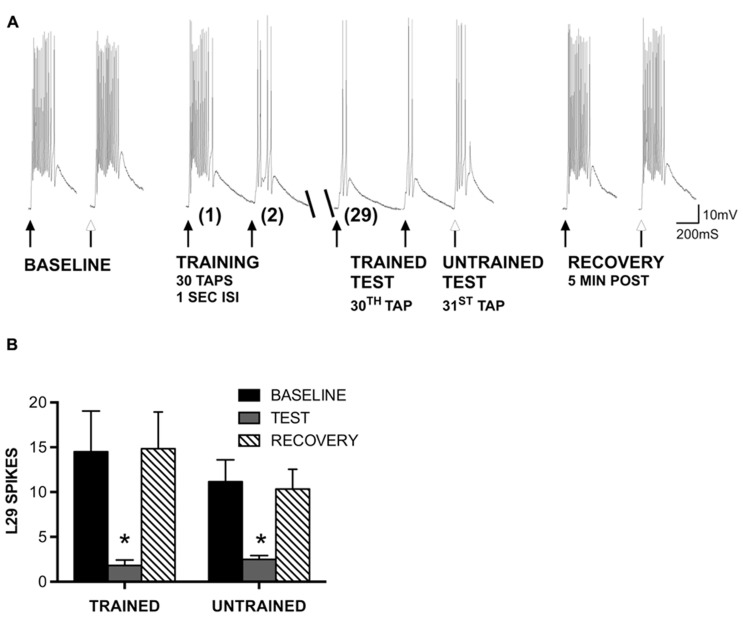
**Generalization of STH in L29 interneurons persists in the absence of inhibition.** **(A)** Siphon tap-evoked responses in L29 interneurons in the presence of 100 μm curare. Two tappers were used (see **Figure 1)**; one for training at a 1 s ISI (TRAINED TEST), the second to test L29 responses 1 s following training at an untrained site (UNTRAINED TEST). Both the trained and untrained sites exhibited reduced responses with training compared to baseline. Responses at both sites recovered 5 min later. **(B)** Quantitative data from six experiments. Both the trained and untrained sites exhibited a significant decrease from baseline (*p <0.05 from baseline using Bonferroni post tests).

**FIGURE 3:**
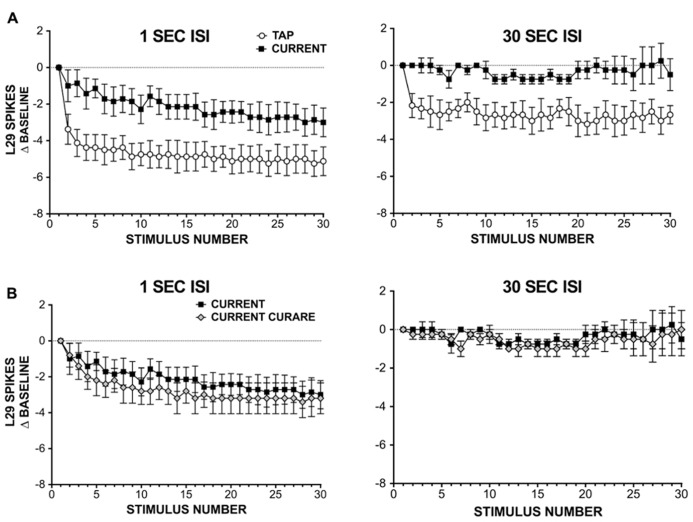
**L29 responses during habituation training with either siphon taps or injected current.** **(A)** Comparison of L29 responses during training with either siphon tap or repeated current injection. Data are expressed as a difference score from the first stimulus. At a 1 s ISI (left), a significant reduction is observed in both conditions, with a greater reduction seen using siphon tap. At a 30 s ISI (right), only siphon taps produced a significant change. **(B)** L29 responses during repeated current injection at ether a 1 s (left) or 30 s (right) in the presence of 100 μm curare. Blocking inhibition has no significant effect on L29 activity.

**FIGURE 4:**
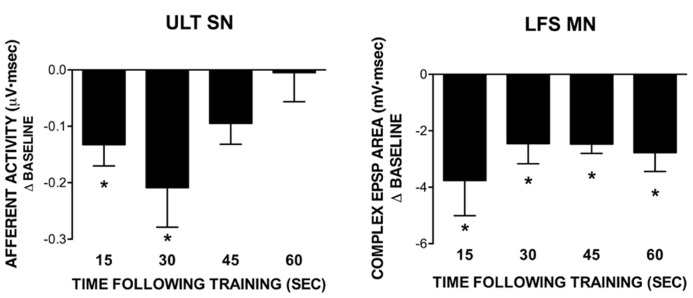
**Recovery of ULT and LFS responses following training with a 1 s ISI.** All data points were obtained in separate experiments. Data represent differences from their respective baseline measures measured at different time points following training; subsequent recovery measures are not shown. The ULT sensory neurons (left) are significantly reduced for up to 30 s following training, with no difference from baseline observed at 45 or 60 s following training. Conversely, LFS MN responses (right) remained significantly reduced from baseline across all time points tested. (*p <0.05 compared to baseline using Bonferroni post tests). Subsequent recovery measures were not different from baseline across all conditions tested.

**FIGURE 5:**
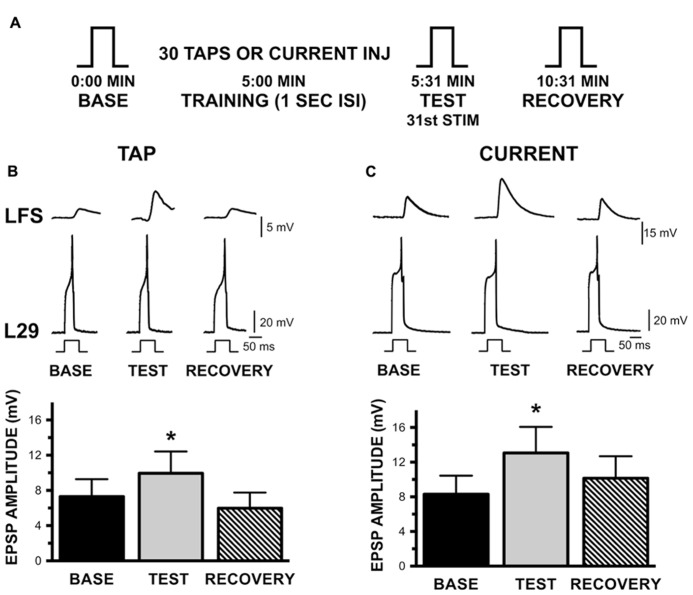
**Augmentation of the L29 to LFS MN synapse with habituation training at a 1 s ISI.** **(A)** Experimental protocol. Steps (BASE, TEST, RECOVERY) indicate activation of a single action potential in L29 in order to measure the amplitude of the EPSP in the LFS MN. 5 min following a baseline measure, training was initiated by either tapping the siphon or injecting current into the L29s. The TEST stimulus occurred 1 s following training by injecting sufficient depolarizing current to initiate a single action potential; RECOVERY measures were obtained 5 min later. **(B)** Measures of the L29 to LFS monosynaptic EPSP in the siphon tap training condition. The top traces illustrate representative physiological measures. All responses are measured by injecting brief depolarizing pulses into the L29s. LFS MNs were hyperpolarized ≈-40 mV below rest, which eliminate the slow potential typically observed at this synapse. Data below the traces represent the average EPSP amplitude across nine experiments. TEST responses were significantly different from baseline. **(C)** Measures of the EPSP in the injected current training condition. Data below the traces represent the average EPSP amplitude across nine experiments. Again, TEST responses were significantly different from baseline. (*p <0.05 from baseline using Bonferroni post tests).

**FIGURE 6:**
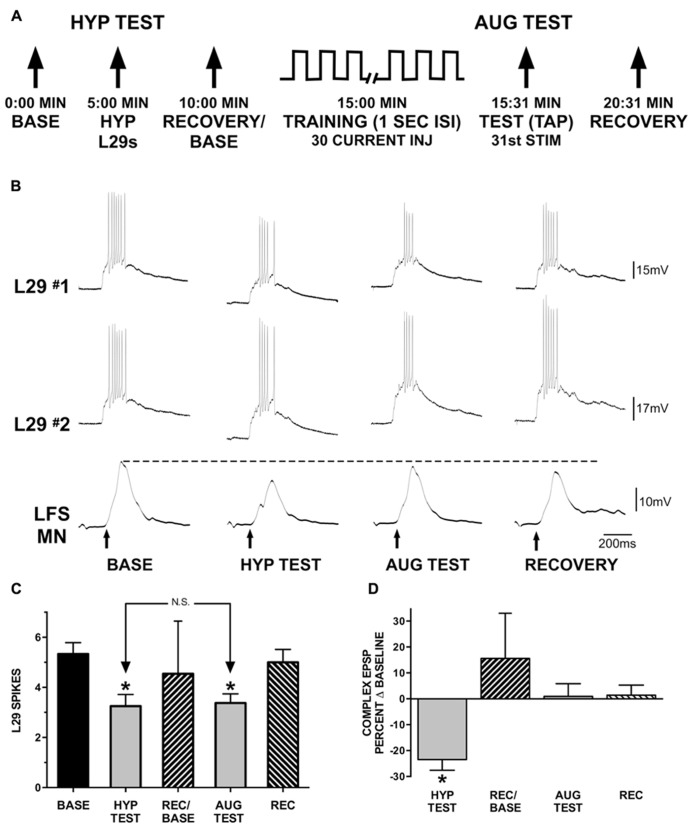
**Augmentation compensates for reduced L29 activity.** **(A)** Experimental protocol. In the first part of the experiment (HYP TEST), tap-evoked L29 activity is decreased by injection of hyperpolarizing current into both L29 neurons. In the second part of the experiment, tap-evoked L29 activity is decreased through repeated current injection at a 1 s ISI into both neurons (intrinsic plasticity; see **Figure 3**). Repeated current injection also augments the strength of the L29 to LFS excitatory synapse (**Figure 5**). **(B)** Representative physiological traces from a single experiment. All responses are evoked by siphon tap (arrows). LFS MNs are hyperpolarized ≈-40 mV from rest to reveal the siphon-evoked complex EPSP. The dashed line indicates baseline-level responding. The dashed line indicates baseline-level responding. In the HYP TEST, the response to tap in both of the L29s is reduced by hyperpolarizing them. This results in a reduction of the tap-evoked LFS MN response. In phase 2, L29’s response to tap is decremented by delivering 30 depolarizing current pulses into both of the L29s. 1 s after these activations, a tap (AUG TEST) is delivered. Note that although the response of L29 to the AUG TEST is decremented to a similar extent as in the HYP TEST, the response of the LFS MN is no longer decremented. **(C)** Summary data of L29 tap-evoked activity from 12 experiments. L29 activity was significantly reduced in both the HYP and AUG tests (*indicates *p* <0.01 from baseline using Bonferroni post tests). There was no difference between the HYP and AUG tests, indicating that L29 activity was reduced to a similar extent in both conditions. **(D)** Measures of the tap-evoked complex EPSP in LFS MNs, normalized to their respective baseline measures so that 0% equals no change. Only measures in the HYP test were significantly different than baseline (statistics performed on raw data; *p <0.01 from baseline using Bonferroni post tests).

Summary data are presented as means ± SEM; probability values reported are two-tailed. Statistical analysis was performed using the program GraphPad Prism (GraphPad Software Inc., San Diego, CA, USA). Analysis of site-specificity of training (**Figure 2**) was performed using two-way repeated measures ANOVA followed by Bonferroni post-tests to compare baseline, test, and recovery trials to each other. To analyze data obtained during training (**Figure 3**), we first determined whether the data for each training curve was best fit by a straight line or a one-phase exponential decay equation using an extra sum-of-squares *F* test. These comparisons took as the null hypothesis that the training curve would be best fit by a straight line, so a significant result indicates that a one-phase exponential decay provides a better fit for the data. Since our previous work determined that training using siphon tap always resulted in a non-linear, one-phase exponential decay (Fischer et al., 2011), this provides a means to assess whether other training methods (e.g., by current injection and/or training in curare) resulted in a different decay function. We determined whether training had an effect by comparing the first and 30th stimulus of a training session with a paired *t*-test. ULT and LFS MN recovery data (**Figure 4**) and L29 augmentation data (**Figures 5 and  6**) was assessed using a one-way ANOVA with the Bonferroni Multiple Comparison Test used for post-hoc comparison of baseline, test, and recovery measures.

## RESULTS

### ACTIVITY-DEPENDENT REGULATION OF L29 EXCITABILITY

In our previous experiments, we found that site-specificity of STH depended upon the neuron-type examined and the training interval used. In particular, L29 excitatory interneurons exhibited generalization of training to untrained stimulus sites at a 1 s ISI but siphon-evoked responses remained training site-specific at a 30 s ISI (Fischer et al., 2011). Here, we examine the potential contribution of synaptic inhibition and intrinsic regulation of excitability to these interval-dependent differences in regulation of the L29s. We first examined whether the generalization of training would persist in the presence of 100 μm curare, which blocks synaptic inhibition within the SWR network (Trudeau and Castellucci, 1993) including from the L30 inhibitory interneurons (Calin-Jageman and Fischer, 2003a). We used the same procedure as in previous work (Fischer et al., 2011), which was adapted from Ezzeddine and Glanzman (2003). Two tappers were positioned on the siphon (**Figure 1A**); one tapper is used for habituation training at a 1 s ISI (trained pathway), the other used to assess site-specificity of training (untrained pathway). A representative example of an experiment preformed in curare is illustrated in **Figure 2A**. Curare was perfused into the recording chamber ten min prior to the start of the experiment. To determine whether curare was effective, we took an initial measure prior to perfusion and compared this to our baseline measure from the “trained” tapper (data not shown). Consistent with previous observations (Trudeau and Castellucci, 1993; Calin-Jageman and Fischer, 2003a), we observed a significant increase of L29 tap-evoked responses in curare (mean difference = 8.0 spikes; *t*-test: *p* <0.05). In the presence of curare, training still reduced responses in the L29s. To determine whether the effects of training were site-specific, a tap was delivered to the second tapper (untrained) as the 31st tap within the same ISI. As in our previous experiments, a reduction in the tap-evoked response was observed. Recovery from training was observed 5 min later.

A summary of six experiments is provided in **Figure 2B**. A two-way ANOVA of all L29 activity data revealed a significant main effect of trial (*p* <0.01), but no effect of training site (*p* = 0.50), indicating similar changes in both pathways. Bonferroni post-tests revealed a significant difference between baseline and test responses for both pathways (trained: *t* = 4.89, *p* <0.01; untrained: *t* = 3.35, *p* <0.01). Recovery measures were significantly different from Test responses (trained: *t* = 5.02, *p* <0.01; untrained: *t *= 3.03, *p* <0.05). These data demonstrate that the generalization of habituation observed in the untrained pathway does not require synaptic inhibition.

Since inhibition was not required for the observed reduction in L29 responses with repeated current injection, we next examined whether intrinsic regulation of L29 excitability could account for these results. These experiments were performed in two parts: in part 1, we repeatedly activated L29s with 30 siphon taps at either a 1 or 30 s ISI; this provides data for comparison to responses obtained with current injection alone. In part 2, we substituted current injection for taps, adjusting the initial current to match the first response observed with siphon tap. Results from these experiments are summarized in **Figure 3A**. At a 1 S ISI, both tap and current resulted in a significant decrease in responding, comparing the 30th response to the first (*n* = 8; tap: *t *= 6.49, current: *t* = 3.81, both *p* <0.01). However, the decay dynamics between the two training methods differed: first, the two training curves were best fit by different types of equations, with current resulting in a linear decay (*F* = 2.52, *p* = 0.11) and siphon tap producing a non-linear, single exponential decay (*F* = 32.77, *p* <0.01). Second, the decrement observed at the 30th stimulus with siphon tap was significantly greater than that from current injection (*t* = 2.62, *p* <0.05). Using a 30 s ISI, only training with siphon tap resulted in a significant decrease in responding (*n* = 6; tap: *t *= 5.39, *p* <0.01; current: *t* = 0.29, *p* = 0.78). The decay during training with tap was best fit with a single exponential decay (*F* = 10.65, *p* <0.01). Taken together, these data suggest that L29 activity alone can regulate L29 tap-evoked responses, but only at a 1 s ISI. The differences observed with siphon tap and current injection at a 1 s ISI likely reflect the added contribution of decreased activity in the ULT sensory neurons resulting from training (Fischer et al., 2011).

The regulation observed with current injection at a 1 s ISI could include a contribution from L30-mediated recurrent inhibition, which has been shown to regulate L29 tap-evoked responses following intracellular activation of L29s (Fischer and Carew, 1993). To examine this, we repeated our experiments using current injection in the presence of 100 μm curare (**Figure 3B**). To compare the two training curves directly, we analyzed the data using a two-way repeated measure ANOVA. For the 1 s ISI, we obtained a significant effect of training (*F* = 15.06, *p* <0.01), but no effect of curare treatment (*F* = 3.22, *p* = 0.10). In contrast, with a 30 s ISI we observed no effect of either training (*F* = 0.92, *p* = 0.99) or curare treatment (*F* = 0.00, *p* = 0.98). These data suggest that inhibition is not required for the regulation of L29 responses during STH. These data also reinforce our results in illustrated **Figure 2** that inhibition is not required for generalization of training, and suggest that intrinsic regulation of L29 activity alone can account for these results.

In previous work, we demonstrated a role for L30-mediated inhibition in extending the time course of regulation following exposure to water turbulence. Briefly, the time course of recovery of siphon-evoked LFS MN responses was longer than that of siphon-evoked ULT responses following exposure to water turbulence (Calin-Jageman and Fischer, 2007). If L30 neurons were hyperpolarized to prevent their activation by turbulence, the time course of recovery of the MNs shortened to match that of the ULTs (Calin-Jageman and Fischer, 2003a). To see if a similar recovery dynamic is present with STH, we measured the time course of recovery of both the LFS MNs and ULT SNs following training at a 1 s ISI. Each post-training measure (15, 30, 45, and 60 s) was obtained in a separate experiment; subsequent recovery measures were obtained 5 min later. Further, experiments for the two cell types were performed separately; siphon-evoked ULT responses were measured using extracellular recordings from the siphon nerve (as in Calin-Jageman and Fischer, 2003a; Fischer et al., 2011), whereas LFS responses were measured using intracellular recordings.

Summary data from these experiments are presented in **Figure 4**, which depicts the difference from baseline for the post-training test measures (recovery measures are not shown). Statistical analyses were performed on raw data using a one-way repeated measures ANOVA. In all experiments measuring post-training recovery of LFS responses (**Figure 4**, right), there was a significant overall effect of training (15 s: *n* = 6, *F* = 5.87; 30 s: *n* = 6, *F* = 9.07; 45 s: *n* = 5, *F* = 31.48; 60 s: *n* = 5, *F* = 9.10; all *p* <0.05). Further, all test responses were significantly reduced from their baseline (pre-training) measures as determined using a Bonferroni post-test (15 s: *t* = 3.31; 30 s: *t* = 4.26; 45 s: *t* = 7.44; 60 s: *t* = 4.08; all *p* <0.05). Recovery measures were not different from baseline. Conversely, for ULT responses (**Figure 4**, left) only the 15 and 30 s post-training measures exhibited a significant overall effect of training (15 s: *n* = 6, *F* = 8.51, *p* <0.05; 30 s: *n* = 6, *F* = 6.98, *p* <0.05; 45 s: *n* = 5, *F* = 3.11, ns; 60 s: *n* = 5, *F* = 0.11, ns). In both the 15 and 30 s recovery experiments, Bonferroni post-tests revealed that test responses were significantly different from their respective baseline measures (15 s: *t* = 3.99; 30 s: *t* = 3.71; both *p* <0.05). In all cases, recovery measures were not different from baseline. Thus, there is a difference in the time course of recovery following training between the two cell types, with an extended time of recovery for the LFS MNs. Since the decrease in LFS MN responses cannot be accounted for by the level of ULT activity, inhibition may play a role in extending the time course of regulation, as we had observed in previous experiments using water turbulence (Calin-Jageman and Fischer, 2003a; Calin-Jageman and Fischer, 2007).

### SHORT-TERM SYNAPTIC PLASTICITY AND RESPONSE NORMALIZATION

These experiments examine how site-specificity of training is maintained in the LFS MNs at a 1 s ISI when it is not maintained in the L29s (**Figure 2**; Fischer et al., 2011). Our hypothesis is that augmentation (AUG), a form of short-term synaptic plasticity expressed by the L29s, may act to maintain a constant level of synaptic input to MNs as L29 activity decreases. L29 neurons have been demonstrated to express AUG following a single activation episode with either tail shock or current injection (Frost et al., 1988; Bristol et al., 2001). As a first step, we examined whether habituation training at a 1 s ISI would also result in AUG.

The basic protocol of these experiments is shown in **Figure 5A**. Each L29 – LFS pair was examined both with activation by siphon taps and by current injection (*n* = 9). The L29 – LFS synapse was examined by eliciting a single action potential in the L29s; the “Test” measure following training was obtained 1 s following training to maintain the training interval. Both activation protocols resulted in significant AUG, as illustrated in **Figures 5B** for siphon tap and 5C for current injection (both *p* <0.01). Post-tests revealed that Test measures were significantly different from Baseline measures in both conditions (tap: *t* = 4.23; current: *t* = 3.50; both *p* <0.01). Recovery was evident 5 min later, as these measures were not different from Baseline (tap: *t* = 2.09; current: *t* = 1.36). These data demonstrate that the L29 to LFS synapse expresses significant AUG with STH. We did not examine whether AUG was also induced with a 30 s ISI.

We next examined the interaction between AUG and the intrinsic regulation of L29 activity as observed in the untrained pathway with STH. These experiments explored the hypothesis that the induction of AUG could compensate for the decrease in L29 activity to maintain net synaptic input to LFS neurons at a consistent level. Experiments (*n* = 12) were performed in two parts, as illustrated in **Figure 6A**: in part one (HYP Test), we examined whether a decrease in L29 tap-evoked responses alone would result in a significant change in the LFS MN complex EPSP. There are five known L29 interneurons within the abdominal ganglion (Hawkins et al., 1981; Frost and Kandel, 1995). While hyperpolarizing a single L29 can have a significant effect on the MN tap-evoked complex EPSP (Fischer and Carew, 1993), this effect required complete inactivation of the L29s, and our goal here was to only reduce activity, not eliminate it. We therefore chose to simultaneously hyperpolarize two L29s in these experiments. We also ensured that both L29s provided synaptic input to the MNs (mean: 5.1 ± 2.5 mV; range = 1.3–9.4 mV). Recovery measures obtained 5 min later were performed with both L29s again at rest; this measure also served as the baseline for the second part of the experiment. In part 2 (AUG Test), we activated both of the L29s with current injection at a 1 s ISI to produce a decrease in L29 activity equivalent to that obtained in part 1. This activation would also induce AUG in the L29s, as was illustrated in **Figure 5**. The question is whether this addition of AUG would compensate for the decrease in L29 activity to maintain the tap-evoked complex EPSP in LFS MNs at baseline levels.

We chose to hyperpolarize both of the L29s to reduce activity in each by approximately two spikes, the average reduction observed at the end of training with current at a 1 s ISI (**Figure 3A**). Summary data illustrating the decrease in L29 activity in these experiments are illustrated in **Figure 6C**. On average, hyperpolarization decreased tap-evoked activity in the L29s by 2.1 ± 1.3 spikes, and training in the AUG test decreased activity by 2.0 ± 1.6 spikes. We analyzed tap-evoked L29 activity across the two parts of this experiment with a one-way ANOVA. We observed an overall significant effect of test condition (*F* = 21.66, *p* <0.01). Post-tests revealed that L29 activity was significantly reduced compared to baseline in both the HYP and AUG tests (HYP: *t* = 6.35; AUG: *t* = 5.97; *p* <0.01 for both). Importantly, there was no difference between the level of reduction comparing the HYP and AUG tests (*t* = 0.38, ns). Recovery measures were not different from baseline.

As shown in **Figure 6B**, reducing L29 activity by just two spikes in the HYP test results in a decrease in the tap-evoked complex EPSP in siphon MNs. Quantitative data from our 12 experiments are presented in **Figure 6D**; data are normalized to their respective baseline measures so that 0% represents no change. On average, the MN complex EPSP was reduced by -23.5 ± 4.1% of baseline with L29 hyperpolarization. Conversely, no reduction was observed when L29 tap-evoked activity was reduced by a similar extent following training with current injection (AUG test); on average, the MN complex EPSP was 0.9 ± 4.8% of baseline. Complex EPSP data from these experiments were analyzed with a one-way ANOVA (this analysis was performed on the raw data, not the data normalized to baseline). We observed an overall significant effect of test condition (*F* = 10.21, *p* <0.01). Post-tests revealed a significant difference between the HYP test and Baseline (*t* = 4.57, *p* <0.01), HYP test and AUG test (*t* = 4.24, *p* <0.01) and HYP test and Recovery (*t* = 4.69, *p* <0.01). No other comparisons reached significance. Since the major difference between the two tests is the induction of AUG, these data demonstrate that this form of short-term synaptic plasticity can compensate for the reduced activity of the L29 excitatory interneurons, effectively normalizing net synaptic input to maintain a constant level.

## DISCUSSION

We have examined STH of the siphon-elicited siphon withdrawal (S-SWR) as a means to characterize dynamic changes within a neural network as it adjusts to accumulating sensory input. Our interest in STH was driven by previous research examining the impact of water turbulence on regulating siphon withdrawal (“environmental regulation”), which produces a continuous and complex form of low-threshold sensory stimulation (Fischer et al., 2000). The comparison was of interest to us because both result in a common behavioral outcome, and both have the net result of optimizing behavioral responding based on the recent history of sensory input (Fischer et al., 2000; Calin-Jageman and Fischer, 2003b). STH provides a means to assess the functional interaction between multiple network processes that include sensory regulation, intrinsic forms of plasticity, and synaptic inhibition under conditions where the temporal patterning of stimuli can be more tightly controlled, and the spatial extent of stimulation is more restricted.

Our focus here was on the L29 excitatory interneurons, which are a major source of excitatory input to siphon MNs (Fischer and Carew, 1993; Frost and Kandel, 1995). Our results show that two activity-dependent processes intrinsic to the L29s, excitability (or intrinsic plasticity: Zhang and Linden, 2003) and a form of short-term synaptic plasticity (AUG) both contribute to SWR network dynamics during STH at rapid (1 s ISI) training intervals, but not at longer (30 s ISI) intervals. Intrinsic plasticity is a commonly observed property in neurons that operates in parallel with synaptic changes to regulate neural networks in an activity-dependent fashion (for reviews see Zhang and Linden, 2003; Benjamin et al., 2008; Sehgal et al., 2013). The L29s have previously been shown to exhibit significant spike frequency adaptation to a single (5 s) current pulse with L30-mediated inhibition blocked by curare (Lieb and Frost, 1997). Our results demonstrate a similar regulation of excitability in the absence of synaptic inhibition to repeated activation during training. This decrease in excitability results in a decreased L29 response to siphon tap, which *if expressed alone* would result in decrease in excitatory input to siphon MNs (as demonstrated in **Figure 5C**).

Our results also demonstrated significant AUG of the L29-LFS MN excitatory synapse at fast (1 s) but not slower (30 s ISI) training intervals. This co-expression of AUG with intrinsic plasticity acts to maintain a stable level of synaptic input to the MNs in the untrained pathway, compensating for the decreased tap-evoked activity of the L29s. A similar interaction has been described in studies of neurons in the crab stomatogastic ganglion utilizing the dynamic clamp technique, where changes in synaptic conductance were shown to compensate for significant variations in intrinsic excitability (Grashow et al., 2010). In a similar manner, we have previously characterized an interaction between AUG and extrinsic regulation of the L29s via the neuromodulator serotonin (5-HT). Bath application of 5-HT results in a decrease in L29 excitability and produces a parallel decrease in the L29 to LFS MN monosynaptic EPSP. Tail shock (which releases 5-HT: Marinesco et al., 2004) produced similar effects, but only in L29 neurons that exhibited little to no action potential activity during the administration of shock. In L29 neurons that responded vigorously to tail shock, significant synaptic enhancement (AUG) was observed that offset the modulatory effects of 5-HT on L29 synaptic transmission (Bristol et al., 2001). Taken collectively, these results reinforce the concept that understanding the contribution of any one form of plasticity at any given neural locus can only be made within the context of other forms of plasticity concurrently active both within single neurons and throughout a neural network (Calin-Jageman and Fischer, 2003a; Marder, 2011).

### MECHANISMS OF SHORT-TERM HABITUATION

The gill and siphon withdrawal reflexes in *Aplysia* provided one of the first model systems to explore synaptic mechanisms underlying habituation. While a primary emphasis in the literature has been on the monosynaptic connection from LE sensory neurons to MNs (for review see Glanzman, 2009), the overall roster of contributing mechanisms will depend on the intensity of the training stimulus, the training interval used, and the site of training on the body surface. An important line of evidence that implicates regulated sensory processing, as a primary overall mechanism is that habituation is training site-specific when both sites are on the siphon itself (Frost et al., 1997; Ezzeddine and Glanzman, 2003; Fischer et al., 2011). Conversely, STH of tail stimulus-elicited siphon withdrawal (T-SWR) appears to be mediated through interneurons (Stopfer and Carew, 1996), and has a requirement for inhibition in the abdominal ganglion that is not shared with STH of siphon-elicited siphon withdrawal (Bristol and Carew, 2005). These observations demonstrate that different network elements can contribute depending upon the particular sensory pathway used for training.

As illustrated in **Figure 1B**, the LE and ULT sensory neurons form parallel pathways from the siphon that differ in their stimulus thresholds (Frost et al., 1997; Hickie et al., 1997; Illich and Walters, 1997; Walters and Cohen, 1997; Calin-Jageman and Fischer, 2007). The relative contribution of each to habituation will depend on the extent a stimulus activates the LE neurons, assuming that the lower-threshold ULTs will be activated by all stimuli capable of activating the LEs (Fischer et al., 2011). This dependence on intensity complicates the ability to isolate the net contribution of the LEs in the absence of ULT activity. It is also difficult to estimate the net ULT contribution to siphon-evoked responses, since monosynaptic EPSPs from these cells have yet to be measured, and the number of cells activated by a stimulus is not known. Further, up to 80% of the siphon-evoked complex EPSP may be mediated through interneurons (Trudeau and Castellucci, 1992). Of the remaining 20%, LEs are estimated to contribute around 5% of the complex EPSP (Hickie et al., 1997; Walters and Cohen, 1997), suggesting that the direct ULT to MN connection also plays an important regulatory role.

The cellular form of plasticity that contributes to habituation appears to differ between the two sensory neuron types. The regulation of the monosynaptic EPSP between LE neurons and MNs with habituation has been well documented (Castellucci et al., 1970, 1978; Castellucci and Kandel, 1974; Byrne et al., 1978b; Byrne, 1982; Cohen et al., 1997; Ezzeddine and Glanzman, 2003). While the LE to MN synaptic efficacy decreases with training, evoked action potential activity of the LEs during training appears to be stable (Byrne et al., 1978a). In contrast, our previous data demonstrated that ULT regulation is based primarily on adjusting the level of activity to match the salient stimulus characteristics of the environment, be it the presence of water turbulence or a particular STH training interval. This change in the level of activity alone could account for training-induced regulation of the SWR network with a 30 s ISI, but not at a 1 s ISI (Fischer et al., 2011) which invokes intrinsic plasticity expressed by the L29s into the regulatory mix, as illustrated in the present work.

The differing cellular mechanisms expressed by the ULTs and LEs raises an interesting possibility on the contribution of these processes to short-and long-term regulation (e.g., within and between sessions habituation). Synaptic depression in LE neurons is restricted to the site of training, and can effectively serve as a long-term “mark” of this experience (Frost et al., 1997; Ezzeddine and Glanzman, 2003). The regulation of ULT activity is well suited to mediate short-term changes, since the level of ULT activity can dynamically adjust to directly reflect the temporal dynamics of tactile stimulation (Calin-Jageman and Fischer, 2007; Fischer et al., 2011). Assuming that the ULTs do not exhibit long-term changes in activity, these mechanisms combined would allow the network to continue to exhibit short-term regulation via regulated ULT activity even when the synaptic efficacy of LE neurons is depressed to reflect the “memory” of the site of habituation training.

## Conflict of Interest Statement

The authors declare that the research was conducted in the absence of any commercial or financial relationships that could be construed as a potential conflict of interest.
